# Factors associated with successful dementia education for practitioners in primary care: an in-depth case study

**DOI:** 10.1186/s12909-019-1833-2

**Published:** 2019-10-28

**Authors:** Cara Sass, Natasha Burnley, Michelle Drury, Jan Oyebode, Claire Surr

**Affiliations:** 1 Centre for Dementia Research, Leeds Beckett University, City Campus, Leeds, LS1 3HE UK; 2 School of Dementia Studies, University of Bradford, Bradford, BD7 1DP UK

**Keywords:** Primary care, Dementia training, Alzheimer’s, Case study, Mixed methods, General practitioners, Quality improvement

## Abstract

**Background:**

With increasing numbers of people in the UK living with dementia, the provision of good quality person-centred care that meets the often complex needs of this population is required. Given the majority of people with dementia live in the community, significant care and support will be provided by primary care services. This means the primary care workforce needs appropriate education to ensure they have the right knowledge, skills and attitudes to meet these care needs. However, little is understood about the most successful approaches to dementia education in this setting.

**Methods:**

An in-depth case study was undertaken in a single primary care organisation with the aim of exploring the impact of a person-centred dementia educational programme, and identify barriers and facilitators to implementation. Data was gathered from a wide range of sources and analysed using Kirkpatrick’s evaluative framework.

**Results:**

Initially, staff learners struggled to incorporate the ‘whole-person’ approach to dementia care, but gained knowledge and confidence through self-directed learning. They reacted positively to the training and appreciated opportunities to learn from peers in other services. They identified improvements in communication and prescribing practices, despite difficulties implementing changes during busy periods. Resultant impact for service users included more timely routine appointments, and positive satisfaction ratings from patients and families.

**Conclusions:**

The findings indicate the perceived value of person-centred dementia education for primary care. Further recommendations for provision in this service setting include tailored programmes designed collaboratively with clinical service providers, and bringing together an interdisciplinary mix of learners to enhance knowledge exchange.

## Background

Dementia is an issue of international concern, with an estimated 50 million people currently living with the condition worldwide [1], including 850,000 in the UK [2]. Globally, timely and accurate diagnosis is a priority [3, 4], and incidence rates have increased steadily over recent years [5–8]. Ensuring staff working within health and social care services have the right knowledge, skills and attitudes to provide good quality care is essential. In the UK, education of the workforce on dementia has been a national priority [9, 10], this includes those working in primary care services. Traditionally the primary care workforce has had little opportunity to access dementia training and research has identified this staff group have a range of education needs in this area [11, 12]. Studies show that training primary care staff about dementia can be effective in improving knowledge [13], attitudes [14] and practice [15, 16]. However, other studies have not found this to be the case [17]. This workforce is often time impoverished and therefore, understanding the characteristics of training most likely to lead to successful outcomes is required.

## Objective

The objective of the primary care case study described in this paper was to understand the impact of a person-centred dementia educational programme on staff reactions, learning, behaviour and outcomes for patients, and to identify barriers and facilitators to provision of effective dementia education in this primary care setting. The specific research questions were:

1. What models of dementia education and training were being adopted? [18]
2. How did staff perceive the training? [19]
3. How did the training impact on staff knowledge, attitudes and practices?
4. How did people with dementia and their family members experience care in sites where staff had received training?
5. What were the specific barriers and facilitators to effective training implementation?

The case study was part of a wider study, the ‘What Works in Dementia Education and Training?’ (What Works?) study [20] which aimed to explore the components of effective dementia education and training for the health and social care workforce and identify barriers and facilitators to implementation. The study comprised three distinct components or ‘work packages’: (i) a review of the literature on the design, delivery and impact of dementia training; (ii) an on-line survey of existing training and education being provided to health and social care staff across the UK and a survey of staff who had undertaken these programmes to examine the effects as well as barriers and facilitators to implementation; and (iii) in-depth case studies of best-practice examples of dementia training across a range of service settings. The ten in-depth case studies included three mental health trust sites, three acute hospital sites, three social care sites and one primary care site. Each type of site had distinctive characteristics due to its context. This paper reports data from the primary care site.

## Method

Case study sites were approached based on their responses to the online training audit. Responses were scrutinised against predetermined training quality criteria developed from findings of the systematic literature review [21] (e.g. component of face-to-face delivery, more than 8 hours’ total duration) and how well the programme content mapped onto the subjects and learning outcomes within the gold standard ‘Dementia Training Standards Framework’ (formerly known as the ‘Dementia Core Skills Education and Training Framework’) [22, 23]. Training had to have been delivered to a sufficient number and range of staff to ensure the opportunity to implement learning to effect practice. There were nine primary care respondents to the audit and six of these outlined programmes that satisfactorily met the quality criteria; however, all six sites were either unresponsive to contact or declined to take part. Survey responses had also been returned by third-party organisations, such as higher education institutions (HEIs) and private organisations, who provided dementia training to health and social care staff. A number of these indicated that they provided dementia training to the primary care workforce. Three of these met the quality criteria. We therefore approached these training organisations and asked them to facilitate contact with sites where they had provided training to one or more members of staff. Using this approach, four general practice (GP) sites were approached to take part. Only one of these sites responded to communication from the study team; they agreed to take part in December 2016.

The case study site comprised a consortia of GP practices covering two Clinical Commissioning Group (CCG) areas in England and more than 360,000 registered patients. The consortium had established a primary care-led Memory Assessment Service (MAS) with the aim of improving timely access to assessment and diagnosis and supporting continuity of post-diagnostic support and care. The service had negotiated a bespoke distance-learning Postgraduate Certificate, addressing dementia assessment, diagnosis and interventions, which was provided by a Higher Education Institution. At the time of the evaluation 24 staff were involved in the service including 11 GPs, 2 diagnostic nurses and 1 pharmacy diagnostician who had received the training, two GPs who were undertaking the training and additional staff not requiring training (e.g. clerical or non-qualified staff).

Impact of training was assessed using the four levels of Kirkpatrick’s evaluative framework [19]:

1. Reactions; reactions to, and opinions of, the training;
2. Learning; the extent of staff learning (including knowledge, attitudes and confidence); based on self-report;
3. Behaviour; self-reported behaviour change in staff;
4. Outcomes; results of training for patients and family members, reported by patients and family members using short survey cards; and self-reported staff outcomes (i.e. stress, burden) based on focus group data.

Data collection took place between February and May 2017. Staff and training facilitator participants were identified and approached via discussion with the organisation’s training lead, who was the person responsible for the initial survey response. All gave written, informed consent to take part.

Data collection consisted of a focus group with 8 primary care staff (6 GPs, a practice nurse and a pharmacist) who had already completed the Postgraduate Certificate training, semi-structured interviews with the training lead (also the service manager) who had been involved with commissioning and development of the training; a training facilitator from the HEI that provided the training, and a mentor, whose role was to oversee development of clinical competence. It was not possible to interview staff members both pre- and post- training as this did not take place during the data collection period. In addition, all 8 participants completed questionnaires concerning barriers and facilitators to implementing their training in practice using an adapted version of the Theoretical Domains Framework of behaviour change [24]. People living with dementia and their families were asked to report their experience of the service using short, anonymous, satisfaction survey cards containing four Likert-scale and open-ended questions. The cards were distributed by GPs and Nurses during MAS clinics, sealed in envelopes and returned to the study team via post. Nine patients and family members completed the cards.

Focus group and interview data for the full set of case study sites were analysed using template analysis, which is an approach to thematic analysis that permits inclusion of a priori themes based on theoretical literature as well as the generation of themes through inductive coding of the research data with data management assisted through the use of NVivo [25]. The major a priori theme headings were imposed (‘top-down’ codes) from our pre-conceived evaluative framework. These were Features of Training (including design and delivery) (Reactions, Learning, Behaviour, Outcomes [19], Barriers and Facilitators to implementation). Relevant sections of data were coded according to this framework. Material under each heading was then analysed inductively to derive sub-themes. A sample of three initial transcripts were simultaneously coded by all in the team, and coding compared and discussed to enable development of an agreed coding framework. A further six transcripts were then coded and consensus meetings held to discuss and agree coding on any areas of difference. Once the final analytic template was agreed all remaining transcripts were coded using this, by one of the research team.

## Results

The results are presented under the top-down headings with sub-themes reflected in the text of each section.

### Features of training

The Postgraduate Certificate (PG Cert) was available nationally to any graduate health professional working in an appropriate role within primary care. It was developed by a University in collaboration with experienced primary care Practitioners from the study site and input from secondary care MAS clinical specialists. The aim was to create specialist primary care practitioners who could undertake dementia diagnosis and prescribing, and signpost to relevant post-diagnostic support, in accordance with the criteria for ‘practitioners with a specialist interest in dementia’, developed by the Royal College of General Practitioners [26]. The support of and accreditation by a Higher Education institution in development and delivery of the programme was felt by the training lead to add credibility and appeal to practitioners by offering a University qualification upon completion.

The programme was delivered via blended learning, with learning materials and exercises predominantly delivered online, alongside face-to-face training days and compulsory practice-based learning within a MAS or similar. Online engagement incorporated real time tutorials and discussion. Learners were assigned a local, experienced, MAS practice mentor (e.g. local MAS consultant) for the duration of their studies. The programme consisted of two 12-week, formally assessed, modules (module one on assessment and diagnosis and module two on post-diagnostic care). The assessments included written assignments, an online examination and development of clinical practice portfolios. The programme was underpinned by a person-centred, whole-person philosophy of dementia care. As the programme was national, participants could be from multiple services.

Table 1 shows the percentage of learning outcomes met by the PG Cert programme, across the covered subject areas within the Dementia Core Skills Education and Training Framework [22]. The Framework contained 14 subject areas across three Tiers. Tier 1 (1 subject) is dementia awareness aimed at all staff working in any role, Tier 2 (12 subjects) is for staff who have regular contact with people with dementia and Tier 3 (13 subjects) for those with leadership roles. Coverage of each subject area was determined from responses to the national survey of training providers (work package 2) which had been completed by the participating HEI. The respondent indicated which individual learning outcomes were covered within each subject area and tier of the Dementia Training Standards Framework. This was converted to a percentage to reflect the proportion of learning outcomes covered by the programme.

**Table 1:** Mapping of learning outcomes to SFH framework modules

Subject area	Tier 1	Tier 2	Tier 3
Dementia Awareness	73%	N/A	N/A
Dementia identification, assessment & diagnosis	N/A	100%	83%
Dementia risk reduction and prevention	N/A	0%	0%
Person-centred dementia care	N/A	25%	0%
Communication, interaction and behaviour in dementia care	N/A	56%	0%
Health & wellbeing in dementia care	N/A	75%	0%
Pharmacological interventions in dementia care	N/A	100%	56%
Living well with dementia and promoting independence	N/A	8%	0%
Families and carers as partners in dementia care	N/A	75%	0%
Equality, diversity and inclusion in dementia care	N/A	0%	0%
Law, ethics and safeguarding in dementia care	N/A	0%	0%
End of life dementia care	N/A	0%	0%
Research and Evidence-based practice in dementia care	N/A	0%	0%
Leadership in transforming dementia care	N/A	0%	0%

### Reactions

Staff reactions to the programme were consistently positive, stating they found the course relevant and engaging and appreciated the flexibility of its organisation. The opportunity to learn from students working in other disciplines and services was also appreciated and this variety of perspectives was felt to contribute to improvements in their service.

“I was really inspired by what was going on in other parts of the country, the dementia friendly communities and all that sort of thing” (FG Participant 002)

Staff indicated the teaching approaches were easy to follow and they felt well supported by academic facilitators. They described the online component as ‘lonely’ at times, but less so when engaging in virtual tutorials and hands-on activities. Some found the volume of work surprising, particularly after a break from formal education. However, programme facilitators believed that the depth was necessary for the postgraduate level qualification. They also stressed the importance of self-motivation in focusing on relevant content. The participating mentor responded positively to the structure of student assessment, which offered flexibility within well-defined competencies.

“I only have good things to say, really. It was very, very broad based reading that was required and I think the mentees had to do a lot of very relevant reading, I would say and very broad … I would say the coursework, really, was very extensive and covered everything we ever needed to address”.

(Practice mentor)

The workload placed considerable demands on the mentors, but they appreciated the opportunity to access their own peer support and reflect on challenges.

### Self-reported learning

The underpinning principles of the PG Cert emphasised holistic understanding of dementia and dementia care. Staff learners reported finding this challenging to grasp, especially during reflective exercises, as this was not a perspective adopted in previous medical training. In terms of diagnostic approaches, staff felt overwhelmed by the perceived complexities of the diagnostic criteria, and the breadth of information they were encouraged to consider in order to make a diagnosis. Staff felt they had gained knowledge in this area especially, and that this had changed their attitudes and approach to assessment and diagnosis in this context.

“[It’s] quite easy to see how people’s perceptions of their approach has changed … I think broadly, one of the main things that came out of that was for a GP of the change in attitudes and the change in perception of their approach.”

(Training Facilitator)

The staff believed that their learning had helped them to signpost patients to other services and treatments. They reported that they had gained confidence in giving timely diagnosis and advice, initiating treatment for their patients and supporting family members, in place of referring to secondary care services.

“And as a resource for your colleagues. They’ll often ask you what to do about somebody who they would probably have had to refer and wait whereas we can say ‘why don’t you try this’ or even prescribe”

(FG Participant 03)

Learners reported gains in dementia-specific knowledge from completing assignments, but felt that improved attitudes and confidence developed from practical application.

### Self-reported behaviour change

Staff were asked about the ways in which training had changed the way they behaved in practice. In particular, they reported an improvement in the way they communicated with people with dementia:

“You might write things down more. Start with much simpler questions. Don’t try and –even when visiting like residential homes, get down on the level of the person. Make sure they can hear you. Just ask one question at a time and give them a chance to answer themselves instead of asking somebody else” (FG Participant 06)

In addition, they gave examples of employing more respectful terminology:

“It’s very tiny, tiny little words, for example before I did the training I would say ‘a demented patient’. Now I wouldn’t dream of saying that, it’s a person with dementia.” (FG Participant 01)

Some also reported giving greater consideration to prescribing practices around anti-psychotic medication and medicines management by patients:

“But it’s even things like ‘once daily’ isn’t it? You know that you’ll go for something that is easier – so that if they’ve got a carer coming in, the carer only has to come in once … stopping unnecessary ones.”

(FG Participant 01)

The strict time limitations placed upon clinic appointments were reported as a barrier to the application of knowledge in practice.

“Yes so while in our consultations we have 45 minutes or whatever it is and you haven’t got time to use all of those things that you learn but it’s sitting there in your head and … of how you interact with people in the assessment clinic I think”

(FG Participant 03)

Learners also reported cascading information to colleagues within the service, meaning their own learning had an impact more widely.

“We share it between ourselves but we also share with other practice colleagues”

(FG Participant 03)

“I did a talk to my GP colleagues, nurses and health assistants and now the last couple of weeks the health assistants have actually come up to see me off their own back and said can we sit in the dementia clinic to actually have an idea about what you do … ”

(FG Participant 06)

### Self-reported outcomes

The training programme provided the primary care staff with the ability to deliver a different service to patients seeking dementia diagnosis, as reported by participants during the focus group:

“I mean I think I felt I was treating them differently post education. I felt more able to talk to them in general and about how they might be managing say their diabetes or something. You hadn’t really thought how hopeless it is if your memory’s not good, trying to remember to eat or to remember your medication or whatever. So I think it made a big difference...”

(FG Participant 01)

They believed their model provided a better service than patients would receive in secondary care using examples such as a waiting time of 3–4 weeks compared to the national targets of 6 weeks.

This perspective of the better quality service in primary care was echoed by the consultant mentor and organisational lead:

“[these patients are] very happy with the service that they have received”

(Practice Mentor)

People accessing the MAS supported these views, reporting consistently high levels of satisfaction:

“I feel I'm cared for well. The team understand the condition and treat me well” (Satisfaction card respondent)

“I am very pleased with the care and attention provided”

(Satisfaction card respondent)

### Barriers and facilitators

A number of barriers and facilitators to completing and implementing training were identified. Barriers included:

- The required volume of work and time commitment, which had been a surprise to some learners;
- Distance learning feeling lonely despite the tutorial opportunities;
- The challenges of adjusting to learning in a different way for learners who had been used to a biomedical rather than person-centred focus during their medical training;
- Lack of time for study and to provide practice mentorship;

Facilitators included:

- Learning with and from a diverse peer group who supported each other;
- The incentives of the University-run course carrying credibility and academic credit;
- Learners’ perceptions of the development of their own knowledge, skills, and capabilities, were an incentive to further learning.

## Discussion/conclusions

The educational programme illustrated in this case study draws upon a number of components highlighted in the wider dementia education literature as likely to lead to effective training implementation for primary care practitioners, such as pairing online learning with face-to-face facilitator interaction, and teaching which incorporates reflective discussion and opportunities for practical, hands-on learning [21].

The programme was generally well received by learners with an identified strength being the collaborative development of the programme between a University and primary care practitioners, meaning it was tailored to practitioner and clinical needs. In spite of the emphasis on person-centred approaches in contemporary dementia training, this approach was new to the majority of learners and, whilst challenging, was described as providing a helpful perspective. This suggests it may be beneficial to introduce a person-centred perspective into medical education [27, 28], with some existing pockets of good practice in evidence [29].

This evaluation revealed numerous positive outcomes in terms of staff knowledge, and both learners and mentors identified specific examples of positive changes in individual practitioner behaviours and service-level approaches. The primary care-led MAS offered by this consortia, underpinned by the training, is innovative and patients indicated they were satisfied with their timely, local access to diagnosis and support. Importantly secondary care practitioners supported this model. Those accessing the service also reported satisfaction with the quality of care that they received.

There are some case study limitations. Although the study team took considerable lengths to identify and make contact with suitable participating sites, only one primary care site consented to take part. However, this particular primary-care based MAS provides a novel example of utilising specialist practitioner training and education to facilitate effective migration of memory services from secondary care settings into primary care. A further limitation arose from inability to collect data from directly observing training sessions, or quantitatively assessing learning through pre- and post-training measures of dementia-related knowledge acquisition, as no training was taking place during the data collection period. Therefore, data relied on self-report and analysis of the hard-copy training materials. Likewise, evidence of behaviour change is derived from self-reports, as direct observations of care practice that were employed in the other case studies in other settings during this research could not be used in this case study due to the nature of the primary care environment.

## Key recommendations

- Developing programmes for primary care through collaboration between training providers and clinical services ensures they meet learning needs;
- Medical training on dementia should include person-centred approaches;
- While there was a clear appeal of accredited provision to primary care practitioners, the workload associated with a formal education programme requires consideration, since this was noted as challenging for both learners and mentors;
- Learning alongside peers from other services and roles may be beneficial for sharing alternative care models and approaches.

## Data Availability

Data may be made available for further research upon request to the authors

## Abbreviations

CCG: Clinical Commissioning Group
HEI: Higher Education Institution
MAS: Memory Assessment Service
PG Cert: Post Graduate Certificate
